# The Association of Food Insecurity and Risk of Mortality: A Systematic Review and Meta-Analysis of Large-Scale Cohorts

**DOI:** 10.3390/nu17111937

**Published:** 2025-06-05

**Authors:** Cyrus Jalili, Seyedeh Parisa Moosavian, Farhang Hameed Awlqadr, Sanaz Mehrabani, Reza Bagheri, Matin Sedighy, Shirley Hodder, Faramarz Jalili, Mohammad Ali Hojjati Kermani, Maryam Zamir Nasta, Sajjad Moradi, Fred Dutheil

**Affiliations:** 1 Medical Biology Research Center, Health Technology Institute, Kermanshah University of Medical Sciences, Kermanshah 6714686698, Iran; cjalili@yahoo.com (C.J.); m.zamirnasta@gmail.com (M.Z.N.); 2 Department of Community Nutrition, Vice-Chancellery for Health, Shiraz University of Medical Sciences, Shiraz 7134814336, Iran; p_moosavian@yahoo.com; 3 Department of Food Science and Quality Control, Halabja Technical College, Sulaimani Polytechnic University, Halabja 46001, Iraq; farhang.hamid.a@spu.edu.iq; 4 Nutrition and Food Security Research Center, Isfahan University of Medical Sciences, Isfahan 8174673461, Iran; sanimehrabani@gmail.com; 5 Department of Exercise Physiology, University of Isfahan, Isfahan 8174673441, Iran; will.fivb@yahoo.com; 6 Department of Clinical Nutrition, School of Nutrition and Food Science, Isfahan University of Medical Sciences, Isfahan 8174673461, Iran; matinsedighy96@gmail.com; 7 School of Health Administration, Faculty of Health, Dalhousie University, Halifax, NS B3H 4R2, Canada; shirley.hodder@dal.ca (S.H.); faramarz_jalili@yahoo.com (F.J.); 8 Clinical Tuberculosis and Epidemiology Research Center, National Research Institute of Tuberculosis and Lung Diseases (NRITLD), Masih Daneshvari Hospital, Shahid Beheshti University of Medical Sciences, Tehran 1983963113, Iran; imhojjati@gmail.com; 9 Research Center for Evidence-Based Health Management, Maragheh University of Medical Sciences, Maragheh 5518654511, Iran; 10 Department of Nutrition and Food Sciences, Maragheh University of Medical Sciences, Maragheh 5518654511, Iran; 11 Preventive and Occupational Medicine, Université Clermont Auvergne, CNRS, LaPSCo, Physiological and Psychosocial Stress, CHU Clermont-Ferrand, University Hospital of Clermont-Ferrand, Witty Fit, F-63000 Clermont-Ferrand, France; fred_dutheil@yahoo.fr

**Keywords:** food insecurity, mortality, cohort, meta-analysis

## Abstract

**Objectives:** Food insecurity (FI) represents a significant global public health issue, yet existing literature presents inconsistent findings regarding its association with mortality risk. This systematic review and meta-analysis aimed to synthesize available evidence to evaluate the relationship between FI and mortality. **Setting:** A systematic search was conducted using the ISI Web of Science, PubMed/MEDLINE, and Embase databases without any date limitation until February 18, 2025. Hazard ratios (HR) and 95% confidence intervals (CI) were pooled using a random-effects model, while validated methods examined quality and publication bias via Newcastle–Ottawa Scale, Egger’s regression asymmetry, and Begg’s rank correlation tests, respectively. **Results:** Findings from 19 studies demonstrated a significant association between FI and increased risk of mortality (HR = 1.23; 95% CI: 1.16, 1.30; *I*^2^ = 83.1%; *p* < 0.001; n = 19). Subgroup analyses indicated a dose–response relationship, with mortality risk increasing by FI severity: mild (HR = 1.16; 95% CI: 1.10, 1.22; *I*^2^ = 0.0%; *p* < 0.001; n = 9), moderate (HR = 1.19; 95% CI: 1.07, 1.31; *I*^2^ = 83.2%; *p* = 0.001; n = 10) and severe (HR = 1.52; 95% CI: 1.25, 1.86; *I*^2^ = 94.9%; *p* < 0.001; n = 10). Additional subgroup analyses revealed a significant association between FI and both all-cause mortality (HR = 1.26; 95% CI: 1.18, 1.35; *I*^2^ = 82.0%; *p* < 0.001; n = 16), and cardiovascular-related mortality (HR = 1.24; 95% CI: 1.11, 1.39; *I*^2^ = 42.8%; *p* < 0.001; n = 7), but not cancer-related mortality. **Conclusions**: Persistent FI appears to contribute to an increased risk of mortality. Hence, it is important to maintain continuity and strengthen current programs aimed at combating FI, which may help reduce FI-related mortality.

## 1. Introduction

Food insecurity (FI) is a condition defined as insufficient access to nutritionally adequate and safe food. According to the U.S. Department of Agriculture (USDA), FI has two levels: (1) Low FI, in which the quality, diversity, or desirability of diet is low, but food intake does not diminish or decreases only slightly; and (2) Very low FI, characterized by decreased food consumption coupled with disrupted dietary patterns [1,2]. FI overlaps with several factors, including social deprivation, increased expenses for housing, racism in economic and social aspects, cost-related aspects, and exceedingly low wages, all of which can lead to FI conditions [3]. However, the primary factor associated with FI in high-income countries is income inequality, rather than a scarcity of food resources. Wealthier countries waste a lot of food that could otherwise be utilized to feed individuals who cannot supply their food requirements [4]. These nations possess more than enough food to adequately feed those who cannot afford it. According to the State of Food Security and Nutrition in the World (SOFI) report, 733 million individuals experienced hunger in 2023, equating to approximately 1 in 11 people worldwide [5]. Moreover, it was estimated that 2.33 million people were confronted with moderate and severe FI globally in 2023 [5].

Food-insecure individuals may select unhealthy foods that are reasonably priced and more accessible compared with healthy, nutritious, dense foods as a result of budget limitations, which may lead to a heightened risk of several diet-related chronic diseases [4]. It should be noted that individuals of lower socioeconomic status and racial/ethnic minorities are more likely to suffer from FI. They also have a higher likelihood of living in environments with limited food resources and are disproportionately affected by diet-related chronic conditions [6]. Multiple previous studies have demonstrated that FI is related to an enhanced risk of several illnesses, including cardiovascular disease (CVD) [7], diabetes [8], hypertension [9], poor sleep quality and quantity [10], anemia [11,12], cancer [13], undernutrition complications [14], and mental related disorders [15]. It is estimated that non-communicable diseases account for 75% of non-pandemic deaths, with the major causes including cardiovascular disease, cancer, chronic respiratory disease, and diabetes. Furthermore, about 82% of non-communicable disease deaths occur in low- and middle-income countries [16]. Therefore, negative health outcomes linked to FI are related to enhanced mortality risk. As FI is associated with numerous chronic illnesses and considering the challenges in effectively managing these illnesses among food-insecure individuals, it is reasonable to expect that FI may lead to a higher risk of mortality. This may be due to the combined impact of poor diet quality, limited access to health care, and the progression of chronic disease [17].

Although a number of prior studies have evaluated the relationship between FI and mortality risk, their findings have been inconsistent [18,19,20,21,22,23,24,25,26,27,28,29,30]. While some studies have found that FI is associated with an enhanced risk of all-cause mortality [22,26,28,31], others have reported no significant relationship between the two [24,25]. In addition, the correlation between FI and specific causes of mortality, including cancer and CVD mortality, was assessed in some studies, which yielded inconsistent results [22,26,27,28,32]. A recent study conducted among U.S. cancer survivors investigated the impact of social determinants of health on mortality risk. After a follow-up period of 249 months, the study found no significant association between FI and all-cause, cancer-related, or non-cancer-related mortality [24]. In contrast, another study using data from the National Health and Nutrition Examination Survey (NHANES) found that individuals experiencing FI had a significantly higher risk of all-cause and cardiovascular-related mortality compared to food-secure individuals [22,33].

FI is a global concern, yet existing studies have yielded inconsistent findings regarding its association with mortality risk. To address these discrepancies, a comprehensive synthesis of current evidence is warranted. The aim of this study is to evaluate the relationship between FI and mortality risk in order to provide clearer and more robust conclusions. The results of the current meta-analysis may inform public health policies and interventions aimed at addressing the impact of FI on population health.

## 2. Methods

This study was conducted in accordance with the 2020 Preferred Reporting Items for Systematic Reviews and Meta-Analyses (PRISMA) guidelines [34]. The research protocol was prospectively registered in the International Prospective Register of Systematic Reviews (PROSPERO) under registration number CRD420251030046.

### 2.1. Literature Search and Selection

A systematic search was performed using ISI Web of Science, Embase, and PubMed/MEDLINE databases without any limitation up to 18 February 2025. The search strategy is illustrated in Supplementary Table S1. Data from grey literature sources, including letters, case reports, reviews, notes, conference abstracts, reports, and short surveys, were obtained from a manual search of references mentioned in original research studies published in one of the noted databases.

### 2.2. Inclusion and Exclusion Criteria

The study inclusion criteria were as follows: a) cohort studies among adult individuals (≥18 years) reporting data on the relationship between FI and the risk of mortality. These studies must report effect estimates in the form of hazard ratio (HR), relative risk (RR), or odds ratios (OR), stating at least a 95% confidence interval (95% CI). Exclusion criteria included the following: (a) research conducted on children and adolescents (<18 years), (b) research with no relevant outcomes, and (c) research with no relevant exposure. Article titles and abstracts, and subsequently, full-text reviews, obtained from database searches meeting the inclusion criteria, were examined by two researchers (SM and CJ). Any disagreements regarding study inclusion/exclusion criteria were resolved by consensus following discussion. The PECOS framework for each study is indicated in Supplementary Table S2.

### 2.3. Data Extraction

Two investigators (S-PM and SM) extracted the following data from articles meeting the inclusion criteria: (a) first author’s name, year of publication, and country of origin; (b) study characteristics; (c) participant characteristics; (d) FI evaluation method; (e) main results; and (f) covariates used for adjustments in multivariate analyses. Any differences of opinion regarding data extraction characteristics were resolved by consensus following discussion.

### 2.4. Quality Assessment

Two researchers (SM and FHA) conducted the quality assessment of each included study by using the Newcastle–Ottawa Scale (NOS) [35]. The consensus of the NOS quality assessment of included studies is reported in Table 1.

### 2.5. Statistical Analyses and Data Synthesis

All statistical analyses were conducted using Stata version 17.0 (StataCorp, College Station, TX, USA). Hazard ratios (HRs) with corresponding 95% confidence intervals (CIs) were used to estimate the overall effect sizes. Effect estimates from the eligible studies were pooled using meta-analytic techniques to provide a comprehensive assessment of the association between FI and mortality risk [36]. The synthesized effect estimates for the current meta-analysis were reported as pooled HR with a 95% CI. Due to anticipated heterogeneity between studies, effect estimates were calculated utilizing the DerSimonian-Laird weighted random-effects model [37]. A pairwise meta-analysis was performed by combining the effect size outcomes for the highest and lowest categories of FI (i.e., highest versus lowest FI categories) to assess the relationship with risk of mortality. The primary results were pooled by applying HR and 95% CI values derived from these comparisons. Heterogeneity in the articles was examined by the Cochran Q and I-squared (*I*^2^) statistics, where the *I*^2^ value was estimated from [(Q − *df*)/Q × 100%]; with Q being the χ^2^ value and *df* the corresponding degrees of freedom. Between-study heterogeneity was considered significant when the Cochran Q statistic was significant (*p* < 0.01) or *I*^2^ > 50%; more specifically, low, moderate, high, and extreme heterogeneity were defined based on the *I*^2^ statistics cut-offs of <25%, 25–50%, 50–75%, and >75%, respectively. Furthermore, to assess potential sources of heterogeneity, subgroup analyses were carried out according to the level of FI (mild moderate, severe), kind of mortality (all-cause mortality, cardiovascular-cause mortality, cancer-cause mortality), age of mortality (premature [before 75 years], mature [after 75 years]), follow-up duration (<10 years, >10 years), number of participants (<10,000, >10,000), region (US, Canada), mean age (<55 years, >55 years, not reported), health status (healthy population, HIV/AIDS patients, cancer patients, cancer survivors, other patients), FI assessment tools (HFSSM scale, Radimer/Cornell scale, other), and COVID 19 pandemic period (before [2019 and ago], during [2019 to 2023], after [2024 and after]). Analyses also considered adjustments for key covariates. In addition, meta-regression analyses [38] were conducted when at least 10 study arms were available to assess whether factors such as sex, body mass index (BMI), smoking status, physical activity, and alcohol consumption influenced the association between FI and mortality risk. Publication bias was evaluated by visual inspection of funnel plots, formal testing by Egger’s regression asymmetry, and Begg’s rank correlation tests [39,40], with outcomes regarded as significant at *p* < 0.05.

### 2.6. Quality of Evidence

The general certainty of evidence across studies was rated using the Grading of Recommendations Assessment, Development, and Evaluation (GRADE) working group guidelines. According to the GRADE evaluation criteria, the quality of evidence was categorized into four levels: high, moderate, low, and very low [41].

## 3. Results

### 3.1. Study Characteristics

**Figure 1:**
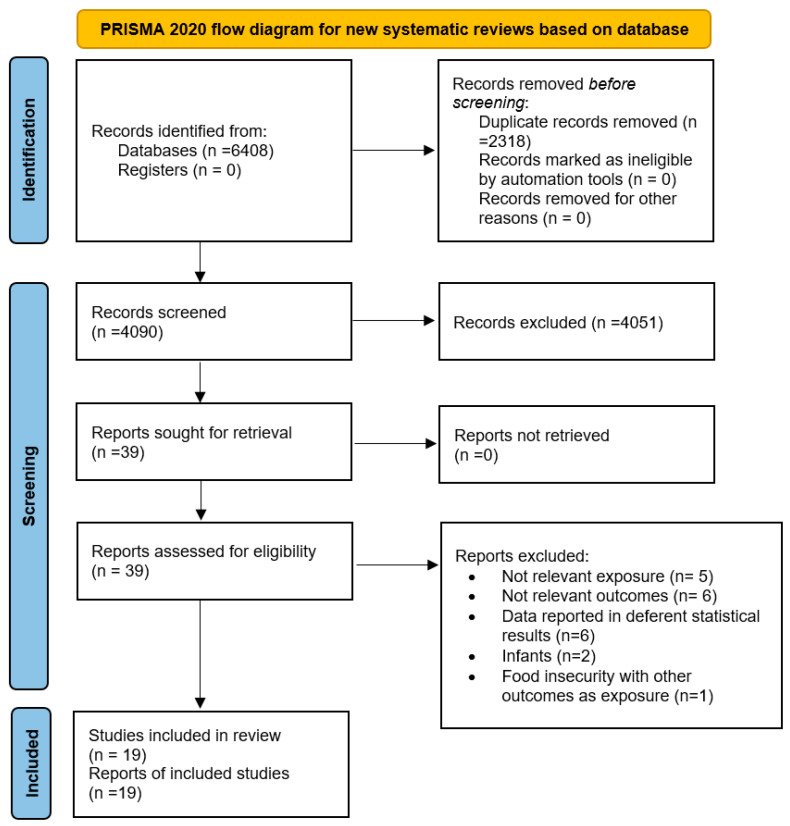
Flow chart of the study selection process. Source: Page MJ, et al. BMJ 2021;372:n71. doi: 10.1136/bmj.n71 [34]. This work is licensed under CC BY 4.0. To view a copy of this license, visit https://creativecommons.org/licenses/by/4.0/, accessed on 1 January 2024.

Our comprehensive search identified a total of 6408 articles. After removing duplicates, 4090 studies remained for evaluation (Figure 1). A title and abstract review led to the exclusion of 4051 articles. Subsequent full-text evaluation of the 39 remaining studies resulted in the exclusion of an additional 20 articles for the following reasons: six studies reported outcomes not relevant to our research scope, five did not focus on relevant exposures, five reported data using different statistical metrics, one focused on infants, and another examined FI as an outcome rather than as an exposure (Supplementary Table S3). Ultimately, 19 articles met our inclusion criteria and were included in the current study [18,19,20,21,22,23,24,25,26,27,28,29,30,32,33,42,43,44,45].

The selected studies (detailed in Table 2) encompassed eighteen cohort studies and one repeated cross-sectional study. The included studies, performed between 2009 and 2025, originated from two countries, including the USA [19,20,21,22,24,25,27,28,29,33,42,43,44,45] and Canada [18,23,26,30,32]. The study-specific, maximally adjusted HRs were reported for 1,443,324 individuals across the selected articles and were pooled for meta-analysis to examine the association between FI and the risk of mortality. Quality assessment using the Newcastle–Ottawa scale classified eighteen studies as high quality [19,20,21,22,23,24,25,26,27,28,29,30,32,33,42,43,44,45] and one study as medium quality [18]. Furthermore, the outcomes indicated that the degree of agreement between investigators for data collection and quality assessment was appropriate (Kappa = 0.803).

### 3.2. FI and Risk of Mortality

**Figure 2:**
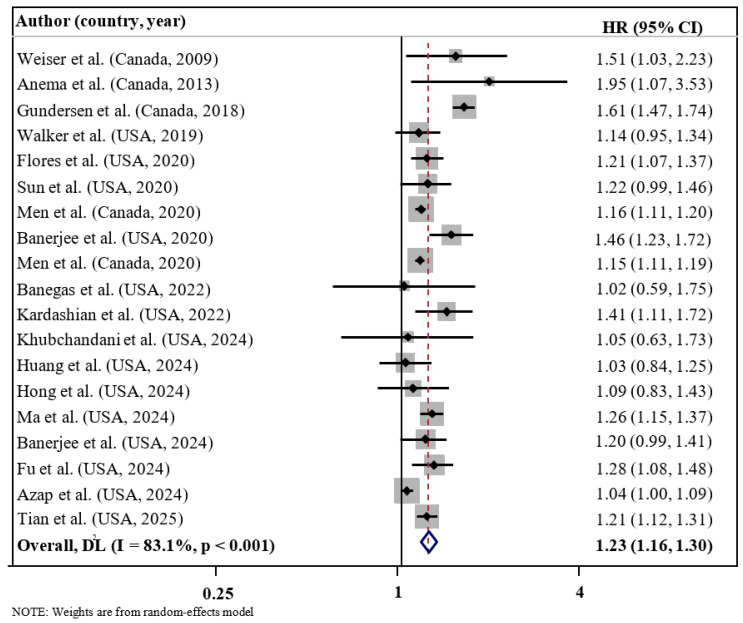
Forest plots demonstrating HR and 95% CI of pooled results from the random-effects models to evaluate the relationship between food insecurity and risk of mortality [18,19,20,21,22,23,24,25,26,27,28,29,30,33,35,45,46,47,48].

The overall analysis results suggested a significant relationship between FI and enhanced risk of mortality (HR = 1.23; 95% CI: 1.16, 1.30; *I*^2^ = 83.1%; *p* < 0.001; n = 19) (refer to Figure 2). A high degree of heterogeneity was detected in selected articles. Subgroup analysis according to the level of FI suggested that the risk of mortality increased with the magnitude of FI from mild (HR = 1.16; 95% CI: 1.10, 1.22; *I*^2^ = 0.0%; *p* < 0.001; n = 9), moderate (HR = 1.19; 95% CI: 1.07, 1.31; *I*^2^ = 83.2%; *p* = 0.001; n = 10), and severe (HR = 1.52; 95% CI: 1.25, 1.86; *I*^2^ = 94.9%; *p* < 0.001; n = 10) (Table 3). Subgroup analysis by cause of mortality demonstrated a significant relationship between FI and increased risk of all-cause mortality (HR = 1.26; 95% CI: 1.18–1.35; *I*^2^ = 82.0%; *p* < 0.001; n = 16) and cardiovascular-related mortality (HR = 1.24; 95% CI: 1.11–1.39; *I*^2^ = 42.8%; *p* < 0.001; n = 7), whereas no significant association was found for cancer-related mortality (HR = 1.04; 95% CI: 0.98–1.11; *I*^2^ = 94.9%; *p* = 0.236; n = 4). Subgroup analysis by geographic region showed a significant association between FI and mortality risk in studies conducted in Canada (HR = 1.19; 95% CI: 1.07–1.32; *I*^2^ = 82.0%; *p* = 0.001; n = 5), but not in those conducted in the United States (HR = 1.07; 95% CI: 0.93–1.25; *I*^2^ = 42.8%; *p* = 0.349; n = 14). Subgroup analysis by health status indicated that FI was significantly associated with mortality risk among the healthy population (HR = 1.25; 95% CI: 1.17–1.34; *I*^2^ = 85.5%; *p* < 0.001; n = 10), HIV/AIDS patients (HR = 1.63; 95% CI: 1.18–2.25; *I*^2^ = 0.0%; *p* = 0.003; n = 2), and other patient groups (HR = 1.30; 95% CI: 1.15–1.48; *I*^2^ = 0.0%; *p* < 0.001; n = 3), but not among cancer patients (HR = 1.04; 95% CI: 1.00–1.09; *I*^2^ = 0.0%; *p* = 0.535; n = 2) or cancer survivors (HR = 1.05; 95% CI: 0.89–1.23; *I*^2^ = 0.0%; *p* = 0.574; n = 2). However, subgroup analyses based on age at mortality, sample size, follow-up duration, mean age, FI assessment, COVID-19 pandemic period, and covariate adjustments did not yield significant differences between groups. Notably, stratification by FI severity, mean age, health status, and cause of mortality contributed to a reduction in observed heterogeneity across studies.

### 3.3. Meta-Regression Analysis

Supplementary Figure S1 presents the results of the meta-regression analyses. No significant effect on the association between food insecurity and mortality risk was found when sex (*p* = 1.00), BMI (*p* = 0.833), smoking status (*p* = 0.545), physical activity (*p* = 0.933), and alcohol intake (*p* = 0.912) were evaluated.

### 3.4. Sensitivity Analyses and Publication Bias

**Figure 3:**
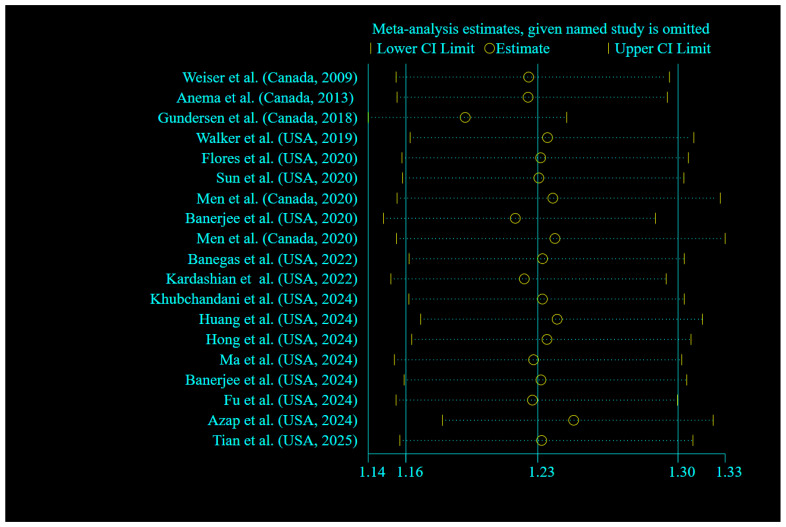
Forest plots showing sensitivity analysis results of the relationship between food insecurity and risk of mortality [18,19,20,21,22,23,24,25,26,27,28,29,30,33,35,45,46,47,48].

**Figure 4:**
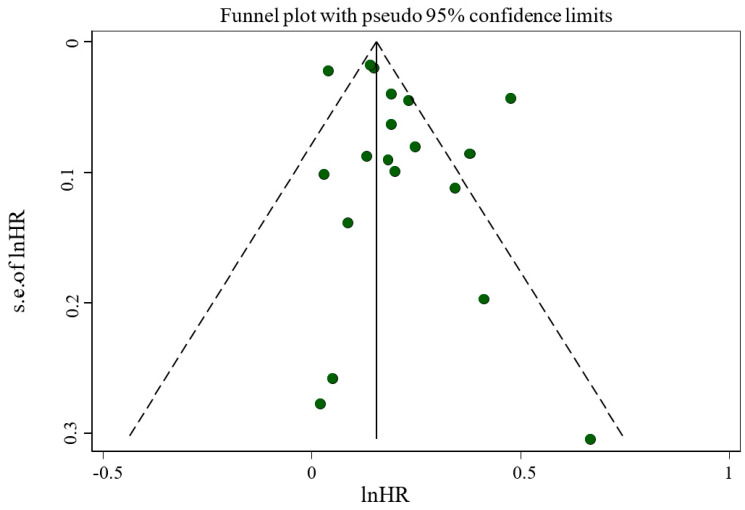
Funnel plot evaluating publication bias among studies reporting risk of mortality.

Sensitivity analysis across the highest to the lowest meta-analysis for FI revealed no significant influence in any single article (Figure 3). No evidence of publication bias was observed among studies related to the association of FI and mortality (*p* = 0.135, Egger’s test; *p* = 0.761, Begg’s). In addition, the funnel plot was symmetrical for the relationship between the FI and mortality (Figure 4).

### 3.5. Quality of Evidence

Using the GRADE approach to assess the quality of evidence, the association between FI and mortality was rated as low, due to concerns related to inconsistency and indirectness (Table 1).

## 4. Discussion

FI is a significant global public health issue, affecting millions of individuals worldwide. Among its numerous health implications, FI has been increasingly recognized for its potential impact on mortality. This meta-analysis systematically synthesized findings from studies examining the association between FI and mortality risk. The pooled results indicated that FI is associated with an elevated risk of mortality. Subgroup analyses revealed that FI was significantly linked to an increased risk of all-cause and cardiovascular-related, but not cancer-related mortality. Regional analysis showed that this association was particularly evident in studies conducted in Canada, while no significant relationship was found in studies from the United States. Furthermore, subgroup analysis based on health status showed a significant association between FI and mortality risk among healthy individuals, HIV/AIDS patients, and other patient groups, but not among cancer patients or cancer survivors. Additionally, a stronger association was observed in studies that adjusted for BMI, suggesting the importance of controlling for this confounder in future studies.

Several factors may explain this discrepancy. The development and progression of cancer are influenced by a complex interplay of variables, including racial and ethnic disparities [46], genetic mutations [47], environmental factors such as smoking [48], nutritional status [49], exposure to environmental chemicals [50], and behavioral factors including mood status [51]. Additionally, access to timely and appropriate treatment, the availability of qualified healthcare providers [52,53], and health literacy levels [54] play a crucial role in determining cancer prognosis. While poor diet quality may contribute to cancer risk, it is important to recognize that cancer mortality is influenced by a wide range of interrelated risk factors.

These factors should be considered when interpreting the results of this work. Supporting this complexity, previous research has shown that cancer-related mortality increases incrementally with a higher cumulative burden of adverse social determinants of health compared to individuals without these disadvantages [24]. In contrast, CVD is a diet-related chronic condition that is more directly affected by FI. Individuals living in food-insecure households often rely on lower-cost, energy-dense foods rather than nutrient-rich options [8]. As a result, FI is associated with increased consumption of sugary foods, processed meats [17], ultra-processed products [55], as well as reduced intake of fruits and vegetables [56]. These dietary patterns are well-established contributors to elevated CVD risk [56,57,58].

Additionally, our analysis revealed a significant relationship between FI and enhanced mortality risk within the Canadian population. In contrast, no such association was observed among studies conducted in the United States. This heterogeneity may be due to several reasons. Previous research has determined that the differences between Canada and the United States in the FI classification scheme have affected the prevalence of household FI. These differences require caution when comparing the prevalence of FI between the two nations and when interpreting the respective study outcomes [59]. A report from Statistics Canada (2022) indicates that 18% of Canadian households experienced FI in the previous year, an increase from 16% in 2021 [60]. In the United States, the Population Survey Food Security Supplement statistics indicated that the prevalence of FI was 12.8% in 2022, an increase from 10.2% in 2021, with 3.8% of individuals experiencing very low FI [1]. The variations in the prevalence of FI among Canadian and United States households could be considered as one of the possible reasons for our findings. It is also possible that FI disproportionately affects high-risk populations in Canada, such as low-income households, where its negative health impacts may be more pronounced. Notably, FI has been reported in over one-third (35%) of Canadian families living below the poverty line [60]. In addition, income inequality, unemployment rates, and poor social support, all of which are key determinants of health-related outcomes [61,62], may vary between food-insecure households in the two regions of the United States and Canada, which could also help explain our finding. In addition to potential measurement and classification differences, variations in public health policies, healthcare accessibility, and underlying population health characteristics (such as the prevalence of obesity, smoking rates, or chronic disease management) may also contribute to the observed disparities between Canada and the U.S. In addition, such differences may be due to differences in the FI assessment tools, varying socioeconomic and healthcare environments, as well as the demographic features of the populations studied. These factors could lead to variability and must be taken into account.

In addition, our findings showed a significant association between FI and mortality risk in healthy individuals, HIV/AIDS patients, and those with other chronic conditions; however, this association was not observed in cancer patients or cancer survivors. Several plausible explanations may account for this lack of significance. First, cancer mortality is influenced by a wide range of complex factors, such as stage at diagnosis, genetic predisposition, comorbidities, and the accessibility and quality of cancer care, which may mask the effects of broader social determinants like FI. Moreover, many of the included studies did not adequately adjust for critical confounders, such as cancer stage, treatment adherence, and nutritional status, all of which can significantly affect survival outcomes.

However, it should be considered that heterogeneity was relatively high between populations and regions. As an example, the *I*^2^ statistic was 70.7% for the Canadian studies and 75.8% for the studies in the US, which indicates high heterogeneity even at the national level. Similarly, heterogeneity for health status varied from 0% in HIV/AIDS cases and cancer survivors to 85.5% among the healthy population. High levels of heterogeneity reduce the precision and confidence in pooled estimates. Moreover, the persistence of the heterogeneity after subgroup and meta-regression analyses indicates the probable effect of unmeasured or residual confounding variables, such as racial or ethnic disparities that were not measured or adjusted for across the included studies.

Furthermore, our findings suggest that the relationship between FI and enhanced mortality risk was evident in studies that adjusted for BMI, but not in those that did not include BMI as a covariate. This may be attributed to the complex and paradoxical nature of FI, which can lead to both undernutrition and over-nutrition, ultimately contributing to overweight and obesity [63]. Individuals experiencing FI may consume energy-dense, nutrient-poor foods as a coping strategy to prevent hunger, thereby increasing their risk of obesity [63], which in turn is a known contributor to elevated mortality risk [64]. Moreover, FI is often associated with obesity-related chronic conditions such as CVD, diabetes, and hypertension, each of which is independently linked to increased mortality [8,65,66]. Therefore, adjusting for BMI in analytical models may help to more accurately capture the true relationship between FI and mortality. Not adjusting for BMI could mask a true relationship because obesity-related comorbidities, including CVD and diabetes, often drive increased mortality risk. These conditions may act as confounders, distorting the observed effects of FI on mortality if not accounted for. In contrast, the absence of a significant association in studies that did not control for BMI suggests that residual confounding by BMI may obscure or distort this relationship.

Understanding the mechanisms underlying the relationship between FI and increased mortality risk is essential for guiding future research and informing effective interventions. Several pathways may explain this relationship. One proposed mechanism is the substitution effect, whereby individuals facing FI are more likely to replace high-quality, nutrient-dense foods with low-cost, energy-dense alternatives that are typically high in simple carbohydrates and unhealthy fats [67].

Several studies support the relationship between FI and low diet quality. A study on community-dwelling adults concluded that diet quality, as assessed using the Australian Recommended Food Score (ARFS), declines with higher levels of FI. Lower ARFS scores reflect a reduced intake of diverse, nutrient-rich foods. Furthermore, marginally food-insecure adults had significantly lower vegetable sub-scale scores. Moderately food-insecure participants had lower scores across all food groups except dairy, while those experiencing severe FI had lower scores on all sub-scales [68].

Similarly, a study utilizing Supplemental Nutrition Assistance Program-Education (SNAP-Ed) data found that fruit and vegetable intake was significantly lower among food-insecure individuals compared to their food-secure counterparts [69]. This study also reported that food-insecure populations exhibited poorer overall dietary habits and lower levels of healthy eating psychosocial precursors. Additionally, individuals at high risk for FI were more likely to consume ultra-processed foods (UPFs) and less likely to consume unprocessed foods compared to those not at risk [70].

A recent meta-analysis supported these findings, indicating that FI is associated with micronutrient deficiencies; notably, 89% of the included studies reported a significant relationship between FI and inadequate nutrient intake [11]. A study conducted on food-insecure households showed a strong correlation between FI and consumption levels below the recommended dietary allowances for many nutrients. Low intake of protein, vitamin C, fiber, vitamin B12, vitamin B5, vitamin A, vitamin B1, manganese, and copper were most strongly associated with the highest rates of FI [71]. Furthermore, another study concluded that with heightened FI, the intake of red meat, poultry, fish, dairy products, fruits, non-starchy vegetables, and nuts decreased, while the consumption of grains, processed meats, potatoes, and sugary foods increased [17].

It is notable that such dietary habits are risk factors for various non-communicable diseases, which are leading causes of death, including CVD, cancer, diabetes, anemia, and stroke. For instance, a previous dose–response analysis study concluded that each 10% rise in UPF intake within daily calorie consumption was related to a 15% higher all-cause mortality risk [72]. Moreover, UPF intake was linked to an enhanced risk of multiple diseases, including CVD, diabetes, obesity, and hypertension [57,73,74,75]. This may be due to their low nutritional quality, including high amounts of saturated and trans fats, sodium, and sugar, coupled with lower content of protein, fiber, and essential vitamins [76,77].

Furthermore, as noted earlier, food-insecure groups have lower levels of fruit and vegetable intake. Considering the inverse relationship between the intake of these food groups and the risk of all-cause mortality [78], the low fruit and vegetable intake among food-insecure individuals may play a role in elevating the risk of some non-communicable diseases, which are leading causes of mortality [79]. The health-protective effects of fruits and vegetables can be explained by their capacity to combat oxidative stress, which is linked to the development of several diseases, including CVD [79]. Rich in antioxidants such as carotenoids and polyphenols, fruits and vegetables help reduce oxidative damage [80,81].

Additionally, minerals like magnesium and potassium, abundant in these foods, offer protective effects against hypertension, diabetes, CVD, and mortality [82,83,84]. Similarly, fiber intake, commonly derived from fruits and vegetables, has been shown to lower all-cause, CVD-related, and cancer-related mortality [85]. This protective effect is thought to arise from several mechanisms, including improved glucose regulation [86], enhanced satiety to reduce obesity risk [87], the production of anti-inflammatory short-chain fatty acids [88,89,90], and decreased cholesterol absorption [91].

In addition, individuals experiencing food insecurity consumed insufficient amounts of nutrients and vitamins. Studies have shown that the risk of all-cause and cardiovascular-related mortality is higher in those with deprived and inadequate nutrient intake compared to individuals with adequate nutrient consumption [92]. Furthermore, a recent meta-analysis demonstrated that individuals experiencing FI have a higher risk of developing anemia and low ferritin levels [11]. The relationship between anemia and increased risk of all-cause, CVD-cause, and respiratory-cause mortality was demonstrated previously [93].

Limited access to nutritious foods among individuals experiencing FI can significantly affect health outcomes by increasing the risk of multiple chronic disorders and hindering the management of diet-related diseases due to poor adherence to specific dietary guidelines [94,95]. FI has also been linked to elevated hemoglobin A1c (HbA1c) levels and a higher risk of developing type 2 diabetes [8,96]. In periods of food scarcity, peripheral insulin resistance may offer a short-term survival advantage by preserving muscle proteins; however, repeated episodes of inadequate food access can worsen insulin resistance and contribute to beta-cell dysfunction in the pancreas [97].

Insulin resistance is related to an enhanced risk of both all-cause and cardiovascular mortality, largely due to its atherogenic properties [31,98] and its contribution to a wide range of morbidities, including metabolic syndrome, hypertension, and dyslipidemia [99,100,101]. Poor nutritional intake also heightens mortality risk by increasing vulnerability to infectious diseases [102], reducing quality of life [101], and prolonging hospital stays [103]. Additionally, FI can be understood through psychosocial mechanisms, particularly its impact on stress, depression, and anxiety, which are known to contribute to both all-cause and cause-specific mortality [31,98]. Chronic stress, for example, is recognized for its role in exacerbating health conditions, including CVD and metabolic disorders. The interplay between FI and psychosocial factors may further elevate the risk of mortality by influencing behaviors that compromise physical health, such as poor dietary choices and reduced engagement in health-promoting activities.

This meta-analysis has several key strengths. First, it comprehensively synthesized data from a 11wide range of observational studies, offering a broad perspective on the relationship between FI and mortality risk. The inclusion of diverse populations enhances the generalizability of the findings. Additionally, subgroup analyses were conducted to explore potential effect modifiers, providing further insight into the factors influencing the relationship between FI and mortality. These strengths collectively contribute to the robustness of the findings, allowing for a more nuanced understanding of how food insecurity affects health outcomes across different populations and contexts. However, some limitations should be considered when interpreting the outcomes. This study demonstrated high heterogeneity in the overall analysis, which may affect the reliability of the results despite the large combined sample size. To address this issue, we conducted extensive subgroup and meta-regression analyses based on potential effect modifiers, including FI severity, cause of mortality, health status, geographic region, FI assessment tools, and age. These analyses indicated that stratifying by certain factors, such as FI severity, mean age, health status, and cause of mortality helped reduce the observed heterogeneity across studies. However, despite conducting subgroup and sensitivity analyses, substantial heterogeneity was observed across some studies. A key contributor to this heterogeneity is variation in FI measurement tools, with many studies using different instruments, most notably the Household Food Security Survey Module, highlighting methodological inconsistencies that limit direct comparisons. Variations in both the number and type of items used to assess food insecurity can result in differences in its classification and the corresponding risk estimates.

Furthermore, due to the observational nature of the selected research, the findings are subject to potential biases, such as selection bias, information bias, and recall bias [104]. In addition, although most studies controlled for certain confounding variables, it is possible that some were overlooked, leading to residual confounding. Therefore, a further limitation is the potential impact of unmeasured confounders. For instance, race and ethnicity, which are important social determinants of health, were not accounted for in most of the included studies, potentially affecting the accuracy of the pooled estimates. Despite these restrictions, the results from the current study provide valuable insights into the significant health risks associated with FI, underlining the importance of addressing this issue in public health policies. Next studies should aim to standardize the measurement of FI, incorporate longitudinal designs to explore causal relationships, and account for additional confounders, such as socioeconomic factors and mental health status, to further refine our understanding of this complex relationship.

## 5. Conclusions

This study found a significant positive relationship between FI and an increased mortality risk. Subgroup analyses further suggested that FI was associated with an elevated risk of all-cause and cardiovascular-related, unlike cancer-related, mortality. Regional differences were also noted, with studies from Canada showing a stronger relationship between FI and mortality compared to those from the United States. Given the role of FI in contributing to adverse health outcomes and increased mortality, it is essential to sustain and enhance current interventions aimed at addressing FI. Policy measures such as financial subsidies for nutritious food may also help reduce FI-related mortality and improve population health outcomes.

## Figures and Tables

**Table 1:** GRADE evidence table for the association between FI and mortality risk. Question: Is there an association between FI and mortality risk in adults? Setting: Adults.

Outcomes	Certainty Assessment							Effect	Certainty	Importance
	№ of Studies	Study Design	Risk of Bias	Inconsistency	Indirectness	Imprecision	Publication Bias	Mean Difference (95% CI)		
Mortality	19	Cohort	not serious	serious	serious	not serious	not serious	1.23 (1.16, 1.30)	⨁⨁◯◯ LOW	IMPORTANT

⨁: Points earned; ◯: Points not earned.

**Table 2:** Characteristics of included studies.

Quality Score	Adjusted Variables	Main Results	Food Insecurity Assessment Method	Mortality Outcome	Population/ Age (Mean ± SD) /(Women/Men)	Study Design/Follow Up (years)/Source of Data/Health Status	Author (Year; Location)
+8/9	Adherence, CD4 counts, and socioeconomic variables	Food insecurity was associated with increased risk of non-accidental mortality	Radimer/Cornell scale	Non-accidental mortality	N: 1119 41 ± 7.79 years (F: 9%; M: 91%)	Cohort 8.3 years BC drug treatment program HIV-infected individuals on HAART	Weiser et al. (Canada, 2009) [30]
+7/9	NR	Food insecurity was associated with increased risk of mortality.	Radimer/Cornell scale	All-cause mortality	N: 354 38.3 ± 6.71 years (F: 16.9%; M: 83.1%)	Cohort 13 years Provincial HIV/AIDS drug treatment Program administrative database HIV-positive injection drug users	Anema et al. (2013, Canada) [18]
+8/9	Age, gender, education level, homeownership, neighborhood-level income quintile, number of children in the household, and number of adults in the household	Food insecurity was not associated with increased risk of mortality	18-item HFSSM	All-cause mortality	N: 90,368 51.3 ± 18.7 years (F: 54.9%; M: 45.1%)	Repeated cross-sectional Canada’s Canadian Community Health Survey Community-dwelling adults	Gundersen et al. (Canada, 2018) [23]
+8/9	Demographics, comorbidities, lifestyle variables, and BMI	Food insecurity was associated with increased risk of all-cause mortality	18-item HFSSM	Mortality	N: 20,918 NR (Mixed)	Cohort 4.5 years NHANES and NDI Community-dwelling adults	Walker et al. (USA, 2019) [29]
+8/9	Age, and gender	Food insecurity was associated with increased risk of mortality, all-cause mortality, and cardiovascular disease mortality	10-item HFSSM	Mortality All-cause mortality cardiovascular disease mortality	N: 25,247 46.5 ± 34.9 years (F: 52.1%; M: 47.9%)	Cohort 10.2 years NHANES linked mortality file Community-dwelling adults	Banerjee et al. (USA; 2020) [33]
+8/9	Age at death, year of death, sex, race/ethnicity, marital status, education, veteran status, and nativity	Food insecurity was associated with increased risk of opioid-related mortality	NR	Opioid-related mortality	N: 11,818 NR (Mixed)	Cohort 3 years All-age death certificate data Community dwelling adults	Flores et al. (USA, 2020) [21]
+8/9	Age, sex, household income, highest education in household, household type, housing tenure, acute care admission to hospital in the past 2 years, number of self-reported chronic conditions, smoker status, and past-year alcohol consumption history	Food insecurity was associated with increased risk of all-cause premature mortality and all causes of death, except cancers	18-item HFSSM	All-cause premature mortality cause-specific premature mortality	N: 510,010 32.5 years (F: 54.5%; M: 55.5%)	Cohort 12 years CCHS and CVSD Community-dwelling adults	Men et al. (Canada, 2020) [26]
+8/9	Age, sex, smoking, alcohol, chronic condition, homeowner, education, household type, and indigeneity	Food insecurity was associated with increased risk of pre-65 mortality	18-item HFSSM	Mortality prior to 65	N: 354,000 58 ± 3.2 years (F: 53.7%; M: 56.3%)	Cohort 7 years CCHS and CVSD Community-dwelling adults	Men et al. (Canada, 2020) [32]
+9/9	Age, sex, race/ethnicity, education, income, smoking status, alcohol intake, physical activity levels, total energy intake, overall diet quality indicated by Healthy Eating Index 2010 score, baseline diabetes mellitus, hypertension, hypercholesterolemia, and BMI	Food insecurity was not associated with increased risk of cardiovascular disease mortality and stroke mortality	10-item HFSSM	Cardiovascular disease mortality Stroke mortality	N: 21,178 57.4 ± 14.5 years (F: 47.9%; M: 52.1%)	Cohort 8.5 years NHANES linked mortality file Community-dwelling adults	Sun et al. (USA, 2020) [27]
+8/9	Sex, sociodemographic, and metabolic risk factors	Food insecurity was associated with increased risk of mortality among participants with NAFLD and advanced fibrosis	18-item HFSSM	All-cause mortality	Adults with NAFLD: N: 4518 48.5 years Adults with advanced fibrosis: N: 1470 65.5 years Total population (F: 51%; M: 49%)	Cohort 4.6 years NHANES linked mortality file Adults with nonalcoholic fatty liver disease (NAFLD) and advanced fibrosis	Kardashian et al. (USA, 2022) [44]
+9/9	Age at diagnosis, sex, race and ethnicity, Elixhauser Comorbidity Index, educational attainment, median household income, Neighbourhood Deprivation Index, type of first-line cancer treatment, cancer type, tumor stage at diagnosis, insurance type, and days between YCLS survey and incident cancer diagnosis	Food insecurity was not associated with increased risk of mortality	Food insecurity was assessed using a single question	All-cause mortality	N: 1151 63.5 ± 18.7 years (F: 59.5%; M: 40.6%)	Cohort 3 years Social risk survey Adults with a new cancer diagnosis	Banegas et al. (2022, USA) [20]
+8/9	Cancer type, cancer stage, urban status, region, marital status, age, sex, and Charlson Comorbidity Index	Food insecurity was associated with increased risk of mortality	10-item HFSSM	Mortality	N: 46,296 75.6 ± 8.9 years (F: 51.5%; M: 48.5%)	Cohort 5 years Surveillance, Epidemiology, and End Results—Medicare database Patients undergoing surgery for colorectal cancer	Azap et al. (2024, USA) [19]
+9/9	Sociodemographic (age, gender, poverty–income–ratio, marital status, and citizenship status) and health-related characteristics (COPD, diabetes, cardiovascular disease, chronic kidney disease)	Food insecurity was associated with increased risk of all-cause mortality among those with hypertension	10-item HFSSM	All-cause mortality	Adults with hypertension: N: 1701 56.5 years (F: 55.8%; M: 44.2%) Adults without hypertension: N: 6462 37.8 years (F: 49.3%; M: 50.7%)	Cohort 8.5 years NHANES linked mortality file among adults with/without hypertension	Banerjee et al. (USA, 2024) [42]
+9/9	Age, sex, race and ethnicity, educational attainment, family income, health insurance, marital status, total number of people in the household, immigration status, smoking, heavy drinking, unhealthy diet, physical inactivity, underlying psychological problems, routine place to go for health care, obesity, diabetes, hypertension, cardiovascular disease, and cancer	Food insecurity was associated with increased risk of all-cause premature mortality	10-item HFSSM	All-cause premature mortality	N: 41,177 47.3 ± 42.6 years (F: 51.3%; M: 48.7%)	Cohort 9.3 years NHANES, and NDI Community-dwelling adults	Ma et al. (USA, 2024) [45]
+8/9	Age, gender, race/ethnicity, education, federal poverty line, marital status, smoking habits, BMI category, diabetes, and CVD	Food insecurity was not associated with increased risk of all-cause mortality, cardiovascular disease mortality, and caner-specific mortality	10-item HFSSM	All-cause mortality Cardiovascular disease mortality Caner-specific mortality	N: 5032 62.5 ± 21.28 years (F: 58%; M: 42%)	Cohort 6.8 years NHANES linked mortality file Cancer survivors	Hong et al. (USA, 2024) [43]
+8/9	Age, gender, and race/ethnicity	Food insecurity was not associated with increased risk of all-cause mortality, caner-specific mortality, and non-cancer mortality	18-item HFSSM	All-cause mortality Cancer-specific mortality Non-cancer mortality	N: 5163 NR (F: 57.7%; M: 42.3%)	Cohort 6.7 years NHANES and NDI Cancer survivors	Huang et al. (USA, 2024) [24]
+8/9	Sociodemographic (i.e., age, gender, education, race, income) and health-related characteristics of participants (i.e., health insurance coverage, smoking, obesity, diabetes, and cardiovascular disease).	Individuals with food insecurity but without CKD did not have a higher risk of mortality. Those with both CKD and food insecurity had a significantly higher risk of mortality	10-item HFSSM	Mortality	N: 13,512 63.6 ± 17.4 years (F: 53.5%; M: 46.5%)	Cohort 9 years NHANES and NDI Adults with chronic kidney disease	Khubchandani et al. (USA, 2024) [25]
+9/9	Age, gender, race/ethnicity, educational level, family income–poverty ratio, smoking status, drinking status, physical activity status, BMI, diabetes, hypertension, heart failure, coronary heart disease, heart attack, stroke, insulin use, hypoglycaemic agents use and HbA1c, high-density lipoprotein, total cholesterol, and systolic and diastolic blood pressure	Food insecurity was associated with increased risk of all-cause mortality	10-item HFSSM	All-cause mortality Cardiovascular disease mortality Cancer mortality	N: 5749 56.1 ± 12.7 years (F: 47.6%; M: 52.4%)	Cohort 8.5 years NHANES and NDI Adults with diabetes	Fu et al. (USA, 2024) [22]
+9/9	NIHS: Age, sex, and race and ethnicity, stratified by survey cycles, employment, income to poverty ratio, home ownership, education status, health care access, insurance status, married or living with partner, smoking status, and supplemental nutrition assistance program enrollment. NHANES: Age, sex, and race and ethnicity, stratified by survey cycles, employment, income to poverty ratio, home ownership, education status, health care access, insurance status, married or living with partner, and smoking status, BMI, hemoglobin A1c, systolic blood pressure, total cholesterol, high-density lipoprotein cholesterol, lipid medications, antihypertensive medications, sleep <6 or >8 h per day, and Patient Health Questionnaire-9.	NIHS: Food insecurity was associated with increased risk of all-cause premature mortality, and premature cardiovascular disease mortality NHANES: Food insecurity was not associated with increased risk of all-cause premature mortality and premature cardiovascular disease mortality	10-item HFSSM 6-item HFSSM 2-item HFSSM	All-cause premature mortality Premature cardiovascular disease mortality	NIHS N: 218,136 44.1 years (F: 51%; M: 49%) NHANES N: 37,027 43.5 years (F: 50.8%; M: 49.1%)	Cohort NIHS and NDI (5 years follow-up) NHANES and NDI (7.8 years follow-up) Community-dwelling adults	Tian et al. (USA, 2025) [28]

Abbreviations: NHANES: National Health and Nutrition Examination Survey; CCHS: Canadian Community Health Survey; CVSD: Canadian Vital Statistics Database; NDI: National Death Index; HAART: highly active antiretroviral therapy; BMI: body mass index. HFFSM: Household Food Security Survey Module, NIHS: National Health Interview Survey; F: Female, M: Male.

**Table 3:** Subgroup analyses of food insecurity and mortality risk (highest vs. lowest category meta-analysis).

Sub-Groups	Number of Effect Sizes	Hazard Ratio (95% CI), P_value_	*I*^2^ (%), P_heterogeneity_	P_between_
**Overall**	19	1.23 (1.16, 1.30), <0.001	83.1, <0.001	
**Level of food insecurity**				**0.032**
Mild	9	1.16 (1.10, 1.22), <0.001	0.0, <0.622	
Moderate	10	1.19 (1.07, 1.31), 0.001	83.2, <0.001	
Sever	10	1.52 (1.25, 1.86), <0.001	94.9, <0.001	
**Kind of mortality**				**<0.001**
All-cause mortality	16	1.26 (1.18, 1.35), <0.001	82.0, <0.001	
Cardiovascular-cause mortality	7	1.24 (1.11, 1.39), <0.001	42.8, 0.106	
Cancer-cause mortality	4	1.04 (0.98, 1.11), 0.236	0.0, 0.444	
**Mean age**				**0.023**
<55 years	8	1.36 (1.21, 1.52), <0.001	87.7, <0.001	
>55 years	8	1.13 (1.06, 1.21), <0.001	60.4, <0.014	
Not report	3	1.15 (1.05, 1.26), 0.002	0.0, 0.339	
**Health status**				**<0.001**
Healthy population	10	1.25 1.17, 1.34), <0.001	85.5, <0.001	
HIV/AIDS patients	2	1.63 (1.18, 2.25), 0.003	0.0, 0.481	
Cancer patients	2	1.04 (1.00, 1.09), 0.074	0.0, 0.944	
Cancer survivors	2	1.05 (0.89, 1.23), 0.574	0.0, 0.547	
Other patients	3	1.30 (1.15, 1.48), <0.001	0.0, 0.535	
**Food insecurity assessment tools**				**0.214**
HFSSM scale	15	1.22 (1.14, 1.30), <0.001	86.2, <0.001	
Radimer/Cornell scale	2	1.63 (1.18, 2.25), <0.001	0.0, 0.481	
Other	2	1.20 (1.06, 1.35), <0.001	0.0, 0.548	
**Age of mortality**				**0.472**
Premature (before 75 years)	3	1.19 (1.14, 1.25), <0.001	31.9, 0.194	
Mature (after 75 years)	16	1.24 (1.14, 1.35), <0.001	83.5, <0.001	
**Follow-up duration**				**0.338**
<10 years	15	1.18 (1.12, 1.22), <0.001	63.4, 0.001	
>10 years	4	1.30 (1.07, 1.58), 0.009	69.5, 0.020	
**Number of participants**				**0.957**
<10,000	8	1.23 (1.11, 1.36), <0.001	26.0, 0.221	
>10,000	11	1.23 (1.14, 1.32), <0.001	89.5, <0.001	
**Region**				**0.268**
USA	14	1.07 (0.93, 1.25), 0.349	75.8, <0.001	
Canada	5	1.19 (1.07, 1.32), 0.001	70.7, <0.001	
**COVID 19 pandemic period**				**0.207**
Before (2019 and ago)	4	1.45 (1.15, 1.84), 0.002	77.3, 0.004	
During (2019 to 2023)	7	1.19 (1.14, 1.25), <0.001	46.7, 0.081	
After (2024 and after)	8	1.16 (1.06, 1.26), 0.001	73.1, <0.001	
**Adjustments**				
**Body mass index**				**0.730**
Yes	7	1.22 (1.13, 1.32), <0.001	89.5, <0.001	
No	12	1.24 (1.17, 1.32), 0.311	0.0, 0.700	
**Smoking status**				**0.470**
Yes	10	1.17 (1.14, 1.20), <0.001	72.4, <0.001	
No	9	1.25 (1.05, 1.48), 0.010	92.5, <0.001	
**Physical activity**				**0.751**
Yes	4	1.24 (1.16, 1.32), <0.001	73.1, <0.001	
No	15	1.22 (1.14, 1.31), <0.001	86.8, <0.001	
**Alcohol intake**				**0.733**
Yes	4	1.20 (1.13, 1.28), 0.001	41.5, 0.163	
No	15	1.22 (1.12, 1.33), 0.001	86.8, <0.001	

Calculated by random-effects model. HFSSM: The Household Food Security Survey Module.

## Data Availability

The original contributions presented in this study are included in the article/Supplementary Materials. Further inquiries can be directed to the corresponding author.

## Supplementary Materials

The following supporting information can be downloaded at: https://www.mdpi.com/article/10.3390/nu17111937/s1, Table S1. Search strategies including the key terms and the queries for each database. Table S2. Description of population, intervention, comparator and outcome (PECOS). Table S3. Reason for exclusion of retrieved articles. Figure S1. Plots showing meta-regression analysis results of the effect of BMI (A), smoking status (B), physical activity (C), sex (D), alcohol intake (E), parameters on the association between food insecurity and mortality risk.
