# Supplementary material for: The Association of Food Insecurity and Risk of Mortality: A Systematic Review and Meta-Analysis of Large-Scale Cohorts
Source: Nutrients. 2025 Jun 5;17(11):1937. doi: 10.3390/nu17111937 (PMC12158141; doi:10.3390/nu17111937)
Supplement: Supplementary file 1 [file nutrients-17-01937-s001.zip › nutrients-3641685-supplementary.pdf]

Supplementary Materials

Supplementary Table S1. Search strategies including the key terms and the queries for each database.

Database February 18, 2025	key terms and the queries
PubMed	Query #1:<br>**(("Food Insecurity"[MeSH Terms] OR "Food Insecurity"[Title/Abstract] OR "Food Insecurities"[Title/Abstract] OR "Food Rationing"[Title/Abstract] OR "Food Supply"[MeSH Terms] OR "Food Supply"[Title/Abstract] OR "Food Supplies"[Title/Abstract] OR "Food Insecurity"[Title/Abstract] OR "Food Insecurities"[Title/Abstract] OR "Food Security"[Title/Abstract])**<br><br>Query #2:<br>**("Mortality"[MeSH Terms] OR "Mortality"[Title/Abstract] OR "Mortalities"[Title/Abstract] OR "Death Rate"[Title/Abstract] OR "Death Rates"[Title/Abstract]))**<br><br>Query #3: #1 AND #2 = 646
Web of Science (ISI)	Query #1:<br>"**Food Rationing**" (Topic) or "**Food Insecurity**" (Topic) or "**Food Insecurities**" (Topic) or "**Food Supplies**" (Topic) or "**Food Supply**" (Topic) or "**Food Security**" (Topic)<br><br>Query #2:<br>"**Mortality**" (Topic) or "**Mortalities**" (Topic) or "**Death Rate**" (Topic) or "**Death Rates**" (Topic)<br><br>Query #3: #1 AND #2 = 1414
Embase	Query #1:<br>'food insecurity'/exp OR 'food insecurity' OR 'food insecurities':ti,ab,kw OR 'food rationing':ti,ab,kw OR 'food supply':ti,ab,kw OR 'food supplies':ti,ab,kw OR 'food security':ti,ab,kw<br><br>Query #2:<br>'mortality'/exp OR 'mortality' OR 'mortalities':ti,ab,kw OR 'death rate':ti,ab,kw OR 'death rates':ti,ab,kw<br><br>Query #3: #1 AND #2 = 2002

Supplementary Table S2. Description of population, intervention, comparator and outcome (PECOS).

**Population**	Adults (≥18 years)
**Exposure**	Food insecurity
**Comparison**	None
**Outcome**	The risk of mortality
**Study design**	Cohort studies

Supplementary Table S3. Reason for exclusion of retrieved articles.

References	Reason for exclusion
1. Pink, Katharina E., Robert J. Quinlan, and Saskia Hin. "Famine-related mortality in early life and accelerated life histories in nineteenth-century Belgium." Proceedings of the Royal Society B 287.1938 (2020): 20201182.<br>2. Maqsood, Muhammad Haisum, et al. "Unfavorable social determinants of health and mortality risk by cardiovascular disease status: findings from a National Study of United States Adults." American heart journal 267 (2024): 95-100.<br>3. Ehrhardt, Matthew J., et al. "Association of modifiable health conditions and social determinants of health with late mortality in survivors of childhood cancer." JAMA Network Open 6.2 (2023): e2255395-e2255395.<br>4. Maqsood, Muhammad Haisum, et al. "Unfavorable social determinants of health and mortality risk by cardiovascular disease status: findings from a National Study of United States Adults." *American heart journal* 267 (2024): 95-100.<br>5. Mostafa, Naydeen, et al. "Malnutrition-related mortality trends in older adults in the United States from 1999 to 2020." *BMC medicine* 21.1 (2023): 421.	Not relevant exposure
6. Roulette, Casey J., and Miriam Kopels. "Perception of uncontrollable mortality risk is associated with food insecurity and reduced economic effort among resource-insecure college students during COVID-19." *American Journal of Human Biology* 36.9 (2024): e24081.<br>7. Yang, Jae-jeong, et al. "Food Security, Serum Carotenoids, and Mortality in US Adults: Findings from the National Health and Nutrition Examination Survey." (2024).<br>8. Hassan, Munum, et al. "Mean temperature and drought projections in Central Africa: a population-based study of food insecurity, childhood malnutrition and mortality, and infectious disease." International journal of environmental research and public health 20.3 (2023): 2697.<br>9. Ogunniyi, Adebayo Isaiah, et al. "Evaluating the Role of Households' Food Security Status and Socioeconomic Determinants on Child Mortality in Nigeria." Child Indicators Research 17.4 (2024): 1687-1714.<br>10. Tamargo, Javier A., and Yenisel Cruz-Almeida. "Food insecurity and epigenetic aging in middle-aged and older adults." Social Science & Medicine 350 (2024): 116949.<br>11. Hassan, Munum, et al. "Mean temperature and drought projections in Central Africa: a population-based study of food insecurity, childhood malnutrition and mortality, and infectious disease." *International journal of environmental research and public health* 20.3 (2023): 2697.	Not relevant outcomes
12. Younossi, Zobair M., et al. "Association of food insecurity with MASLD prevalence and liver-related mortality." Journal of Hepatology 82.2 (2025): 203-210.<br>13. Gondi, Keerthi T., et al. "Health of the food environment is associated with heart failure mortality in the United States." Circulation: Heart Failure 15.12 (2022): e009651.<br>14. Endo, Yutaka, et al. "The impact of county-level food access on the mortality and post-transplant survival among patients with steatotic liver disease." Surgery 176.1 (2024): 196-204.<br>15. Ghio, Michael, et al. "Firearm Homicide Mortality is Linked to Food Insecurity in Major US Metropolitan Cities." *The American Surgeon*™ (2025): 00031348241281848.<br>16. Twine, Wayne, and Lori Mae Hunter. "Adult mortality and household food security in rural South Africa: Does AIDS represent a unique mortality shock?." Development Southern Africa 28.4 (2011): 431-444.<br>17. Wang, Stephen Y., et al. "Food insecurity and cardiovascular mortality for nonelderly adults in the United States from 2011 to 2017: a county-level longitudinal analysis." *Circulation: Cardiovascular Quality and Outcomes* 14.1 (2021): e007473.	Data reported in deferent statistical results (β coefficient, Interquartile Range, Percent change)

**Campbell, Ashley A.,** et al. "Relationship of household food insecurity to neonatal, infant, and under-five child mortality among families in rural Indonesia." Food and nutrition bulletin 30.2 (2009): 112-119..<br>18. Cassidy-Vu, Lisa, Victoria Way, and John Spangler. "The correlation between food insecurity and infant mortality in North Carlina." Public Health Nutrition 25.4 (2022): 1038-1044.	Infants
19. Khubchandani, Jagdish, et al. "Food Insecurity is Associated with a higher risk of mortality among Colorectal Cancer survivors." Gastrointestinal Disorders 6.2 (2024): 461-467.	Food insecurity with other outcomes as exposure

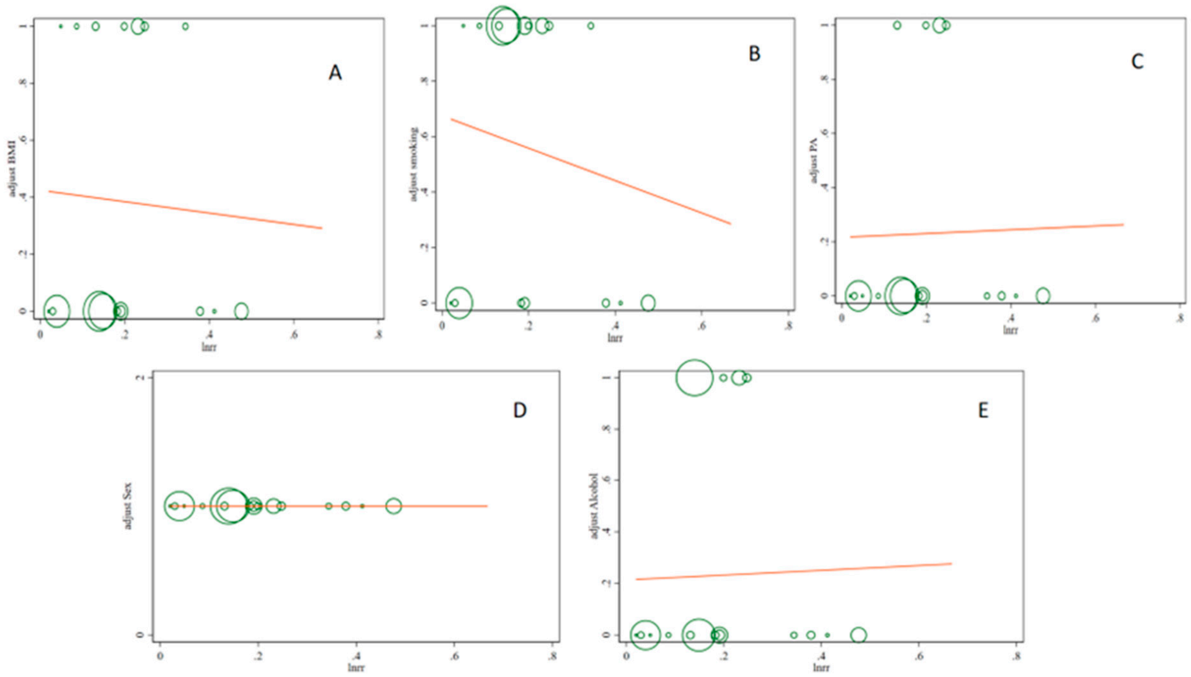

Supplementary Figure S1. Plots showing meta-regression analysis results of the effect of BMI (A), smoking status (B), physical activity (C), sex (D), alcohol intake (E), parameters on the association between food insecurity and mortality risk.
